# Axial Spondyloarthritis and Cigarette Smoking

**DOI:** 10.2174/1874312901711010053

**Published:** 2017-05-31

**Authors:** Irène Kona Kaut, Fatima Ezzhara abourazzak, Essouri Jamila, Florine Awassi Sènami, Desiré Diketa, Harzy Taoufik

**Affiliations:** 1 Sidi Mohammeh Ben Abdellah University, Faculty of Medicine and Pharmacy, Fes, Morocco; 2 Department of Rheumatology, CHU Hassan II, Fes, Morocco; 3 Departement of Epidemiology, CHU Hassan II, Fes, Morocco; 4 Departement of Rheumatology, Abdelmalek Essaadi University, Faculty of Medicine of Tangier, Fes, Morocco,

**Keywords:** Axial spondyloarthritis, Smoking, Severity

## Abstract

**Background:**

Smoking is one of the main environmental risk factors involved in several rheumatic diseases according to ACR 2014, it is included among the risk factors for severe axial spondyloarthritis.

**Objectives:**

The objective is to study the association between smoking and the activity of the disease, the functional impact and the severity of the axial spondyloarthritis.

**Methods:**

It is a transversal study with a descriptive and analytical aim, during the period between January 2014 and December 2015 conducted in the department of rheumatology at the CHU Hassan II of Fes.

The data was recorded and analyzed using SPSS v20 univariate and bivariate analysis

A value of p <0.005 has been used to identify factors associated with smoking.

**Results:**

The study included 214 patients, 130 men and 84 women. The mean age was 39.77 ± 13.06 (16-68) years with an average disease duration of 7.97 ± 6.4 (2-35) years.

The prevalence of smoking in patients with spondyloarthritis was 36%.

According to the univariate and bivariate analysis: Smoking was associated with the male sex (p≤0.0001), with a functional impairment BASFI (p = 0.038) and activity BASDAI (p=0.004) and ASDAS CRP, (p=0.036).

Multivariate logistic regression analysis suggested that smoking was associated with male sex and the severity of the disease.

**Conclusion:**

Our study suggests that there is a significant association between smoking and male sex and the severity of the disease.

## INTRODUCTION

Axial spondyloarthritis is one of the chronic inflammatory rheumatic diseases characterized by spinal and sacroiliac joint syndrome but also enthesic syndrome and peripheral joint syndrome [1].

There are radiagraphic and non-radiographic axial spondyloarthritis.

It is considered to be due to a complex interaction between environmental factors such as viruses, bacteria, tobacco, and genetic factors among others HLA B27 Antigen, which can lead to immune reactions and cause different rheumatic disorders [2, 3]. The most contentious environmental factor recently is smoking.

Tobacco being identified as one of the most serious health problems is the subject of several studies on its influence on several diseases like rheumatic diseases including rheumatoid arthritis (RA) [4, 5], psoriatic arthritis and Systemic lupus erythematosus (SLE) [6, 7].

The mechanism of action of tobacco in these different pathologies would be to induce inflammation by various means: increase of pro-inflammatory cytokines, decrease of anti-inflammatory cytokines, promotion of Th1 cells and activation of macrophages.

Several studies have examined the influence of smoking on the severity of rheumatoid arthritis (RA) [7-14] and it has recently been shown that smoking induces rheumatoid arthritis in favor of citrillunization. 

However, relatively few studies have focused on AS and less have yet specifically examined the effect that smoking has on functional disease activity (BASFI), and the quality of life of patients with d AS. [13-18].

We first showed in 2012 in 647 patients the DESIR cohort, the association between smoking and early spondyloarthritis, its clinical activity (BASDAI), and a more serious defiance.

 They also demonstrated the influence of tobacco on inflammation of the spine and sacro-iliac joints, hence the more severe structural damage judged on the mSASSS4 score 3.

Smoking could be an important environmental factor in the process of inflammatory rheumatic disease including axial spondyloarthritis (AS).

Environmental and lifestyle factors such as smoking may override the hereditary factors that influence the functional state of a person in AS. This would increase the probability of morbidity.

In addition, smoking appears among the factors associated with an increased risk of advanced disease, higher activity and early onset of inflammatory back pain in young patients [17, 18].

The aim of our study was to evaluate the association between tobacco and the various clinical and biological characteristics of axial spondyloarthritis AS such as inflammatory biological syndrome (SV and / or CRP), disease activity by BASDAI and ASDAS (CRP and VS),

The functional impact, the spinal stiffness (reduced physical mobility) and finally the severity of axial spondyloarthritis.

## PATIENTS AND METHODS

### Population:

Our study included 214 patients with AS selected according to modified New York criteria or ASAS criteria, seen in consultation or hospitalized in the department of rheumatology CHU Hassan II of Fez, during the period extending from January 2014 to December 2015.

 Inclusion criteria: primary axial X-ray and non-radiographic spondyloarthritis.

Exclusion criteria: spondyloarthritis associated with psoriasis, IBD as well as reactive arthritis. Inflammatory disease other than AS, malignant tumor pathology, central nervous system involvement, chronic renal failure, and hepatopathy.

### Methods:

 This is a cross-sectional study with a descriptive and analytical purpose, during the period between January 2014 and December 2015 conducted in the department of rheumatology at the CHU Hassan II of Fez.

1. Sociodemographic data of patients: Identity, age, sex, place of residence (urban or rural), level of education (illiterate, primary, secondary, tertiary), occupational activity, ethnicity, Dyslipidemia, alcohol or other), current and past tobacco consumption (at least 3 years) was collected.
2. Characteristics of the SA: We analyzed: duration of development, modified New York diagnosis criteria or ASAS, axial, peripheral and enthesic manifestations, disease activity (BASDAI: Bath Ankylosing Spondylitis Disease Activity Index [16, 19] and ASDAS: Ankylosing Spondylitis Disease Activity Score, its severity defined by the presence of at least one of these criteria: (young age of onset, coxite, uveitis, Refractory NSAIDs, Inflammatory Syndrome (ESR and CRP taken together), and its functional effect (BASFI: Bath Ankylosing Spondylitis Functional Index) in a valid Arabic version [15, 20]. A disease was considered active if BASDAI ≥4.

Concerning ASDAS:

- Low activity if ASDAS <1,3
- Moderate activity if 1,3 ≤ASDAS <2,1
- High activity if 2,1 ≤ASDAS <3,5
- Very high activity if ASDAS ≥3,5

A functional repercussion was present if BASFI≥ 4. The scores have a final range from 0 to 10. If it is greater than 4 is considered active.Later, physical examinations were performed to determine the patient's physical mobility, Lumbar flexion (modified Schober index) and finger-to-floor distance (FFD)concerning the Lumbar stiffness; thoracic expansion (TA)concerning the dorsal stiffness and occiput-to-wall (OWD) distance,Menton-sternum in flexion and extension, distance Menton-acromion, Distance tragus -acromion, Distance occiput-wall and Distance C7- wall concerning the cervical stiffness.The presence of a sedimentation velocity> 30 mm at the first hour and (ESR) / or a high CRP was considered as an inflammatory biological syndrome. An axial spondyloarthritis was considered as severe by the presence of at least one of S: coxitis, uveitis, systemic inflammation, young age of onset, NSAID resistance and smoking which was assessed by specifying when the smoking was done: Current smoker versus past Smoker's at least 3 years.

Statistical analysis was performed using the SPSS statistical module. Fisher's exact test was used to analyze group differences. Correlations between the variables were determined by the Spearman rank correlation test. A multivariate logistic regression analysis was performed to calculate the odds ratio (OR) of various clinical parameters and the P values ​​were considered significant if they were less than 0.05.

### Results:

Male predominance was observed in our sample with 130 men (62.2%) and 80 women (37.8%), and a sex ratio (M / F) = 239.3% of the patients were diagnosed According to the modified New York criteria and 60.7% according to the ASAS criteria. The mean age was 40.29 ± 13.06 (16-68) years with an average disease duration of 7.97 ± 6.4 (2-35) years. The prevalence of smoking in patients with spondylitis was 36% Fig. (**1**). These AS patients were divided into two subgroups: Smokers (current smokers and past smokers) and non-smokers. There was no difference statistically (P = 0.618) and duration of illness (p = 0.096). There was a statistically significant difference between smokers and non-smokers e with regard to the activity of the disease according to The BASDAI index (p = 0.004) and the ASDAS CRP index (p = 0.036). 

Among the parameters of physical mobility and other clinical features of AS, there was a statistically significant difference in dorsal stiffness (p = 0.003) and cervical stiffness (p = 0.031). No association between tobacco and: VS p = 0.824 and CPR: p = 0.084 VS. It was noted that functional repercussion (p = 0.038) and the severity of the disease p = ≤0.0001 (presence of at least one severity factor) were associated with smoking (Table **1**).

We were able to analyze also other clinical manifestations that may affect patients with axial spondyloarthritis and considered as criteria of severity of the disease. The presence of coxite (p = 0.038) and the biological inflammatory syndrome (VS and CRP: p = 0.023) were associated with tobacco (Table **2**).

Using multivariate logistic regression analysis, after removing the confounding bias, according to the odds ratio for the relationship between smoking status and different clinical parameters we found that smoking was associated with the male sex (OR = 80.90;95% CI [17.95 - 364.64],P = ≤ 0.001), activity according to BASDAI (OR = 2.545; 95% CI, [0.973 ─ 6.660], p =0.059), and functional impairment BASFI (OR 2.357,95% CI [ 0.935 ─ 5.941],p = 0.06) and the severity of the disease (OR = 6.773, 95% IC [1,248 ─ 36,754] , p = 0.027). (Table **3**).

## DISCUSSION

Our study showed that smoking was associated with the male sex, the activity of the disease by the calculation of ASDAS CRP and BASDAI, functional impairment, spinal stiffness and severity of AS according to different criteria SA Which will be a little more detailed later on.

- The prevalence of smoking in the spondyolarthritic patients of our series was 36% Fig. (**1**). This is close to the prevalence found in the literature reported by various studies that varies between 37.2% and 76% [3-4, 12-16, 21-22].
- We found that tobacco is significantly associated with the male sex in our study while in the literature there was no significant relationship: we find almost as many men And women smokers. This can be explained by Moroccan habits, since smoking is considered a taboo among women, the latter do not admit that they smoke, so that we can not effectively affirm that there are more men who smoke than women In Morocco.
- Concerning the physical mobility of spondyloarthritis patients in our study, we observed spinal stiffness at the cervical level which was statically significant by the measurement reduced the distance Menton-sternum in flexion and extension, distance Menton-acromion, Distance tragus -acromion, Distance occiput-wall and Distance C7-Wall) and at the dorsal level also which was statically positive by measuring the amplitude which was reduced chest. In general spondyloarthritis patients become stiff over time, depending on the course of the disease especially if not properly managed including rehabilitation. Our study shows that this evolution towards stiffness is more important in the current smoking spondyloarthritic patients but not in the old smokers ATCD, which implies that the tobacco accelerates through the inflammation this process of ossification which will end up The ankylosis of the SPA responsible for the stiffness. This has been found in the literature where several studies have described the harmful influence of smoking on several characteristics of axial spondyloarthritis such as: A British cohort of 53 AS patients, By Averns HL *et al.*: They demonstrated that smokers had reduced mobility through the measurement of finger ground distance, Schober test, total spinal motion, and distance between the occiputal walls.

Reflected the spinal stiffness of the vertebral column. - A study of 48 AS patients in Turkey showed that there was a significant decrease in the modified lumbar Schober test and chest expansion, and an increase in hand-to-ground distance in non-smokers [15].

- In our study, there was no significant association between ESR, CRP and tobacco. It was not relevant in our study that the parameter that takes into account the severity of the disease is the biological inflammatory syndrome (ESR and CRP taken together) was significantly elevated in smokers with AS compared with non Smoking, this is fully explained by the fact that smoking affects inflammation by various mechanisms mentioned above, this could lead to the severity of the disease by exacerbating the inflammation. Several studies have identified smoking as a factor in aggravation of inflammation in axial spondylitis [3, 12, 21] and inflammation depends on the cumulative dose of tobacco.

A study of 48 AS patients in China showed That the parameter of inflammatory syndrome was higher in smokers with AS than in non-smokers, these were statistically proved [3-6].

In this same Chinese study, smokers of cigarettes with AS With t high inflammatory biological syndrome had reduced physical mobility. In addition, the increase in smoking intensity among smokers Of AS is associated with several disease outcome criteria, including the functional impairment and physical mobility that leads to spinal stiffness. This was comparable to our study where there was a greater functional repercussion in patients with smoking spondyloarthritis than in non-smokers and this was statistically significant after the logistic regression analysis.

AS, our study showed that current smokers had high activity compared to non-smokers and former smokers although it was statistically significant in bivariate analysis but this was not statistically significant after the logistic regression. The more the patients smoke the more they kept the disease active because the tobacco acts by causing the inflammation from where staying smoker keeps the inflammation but it was not possible to say whether it depended on the intensity of the smoking It was not possible to collect data on the basis of number of packets per day or per week to have the number of packets accumulate over the year.This seems to be the object of several studies in the literature that have become more Based on the influence of smoking on the activity of spondyloarthritis, in particular the effect of smoking on the activity of spondyloarthritis [17, 18, 23-27]. They demonstrated that smoking AS patients had significantly higher activity by the BASDAI scores [3, 14-24].

Smoking has been associated with increased disease activity and radiographic severity in established AS in some studies [17]. Current but not previous smoking or smoking intensity has been recently reported to be a major risk factor for incident AS, supporting the hypothesis that smoking may be causally related to the development of AS [28].

Kaan and Ferda observed the effect of cigarette smoke in smoking cessation. On the activity of the disease and found that smokers had more active ankylosing spondylitis (BASDAI) than non-smokers [15]. Similarly, Reed et al found that current smokers but not those with ATCD had significantly higher BASDAI results [14]. A series of cohorts showed that smoking was associated with a higher disease activity (p = 0.003), and a functional repercussion of spondyloarthritis.In the literature it has been found that several studies have indicated that smoking has an effect Negative on different functional characteristics in AS [14, 15, 29]; Which may even have an impact on radiographic lesions that are more serious thing that has not been the subject of our study since we did not carry out the evaluation on MRI.

Our study showed that there was a Significant association between tobacco and the severity of the disease This was confirmed after logistic regression, this being because the current smoking patients had at least one severity criterion including coxite, young age of onset and inflammatory biological syndrome which were also Significantly associated with tobacco. Different studies agree with our results: they revealed that smoking has a dose-dependent effect with severity of disease (AS) by studying several severity criteria [30-37].

## CONCLUSION

In our study we observed that smoking is associated with the activity of the disease, spinal stiffness in Moroccan patients with spondyloarthritis. Smoking is also associated with the severity of the disease by the presence of at least one of the criteria for the severity of the disease in addition to the inflammatory biological syndrome and coxite in patients with AS.

Several studies in the literature have obtained the same results found regarding the impact or influence of tobacco on spondyloarthritis.

However, the impact of smoking on the AS disease process cannot be established on such an observational cross-sectional study with a descriptive and analytical purpose. To assess the association between smoking and the outcome of the disease, a longitudinal follow-up study of disease activity, functional capacity and physical mobility is necessary. Since smoking is a public health problem, a devastating environmental factor in health; so smoking cessation is an important recommendation for AS patients to avoid long-term deterioration of the disease.

## Figures and Tables

**Fig. (1):**
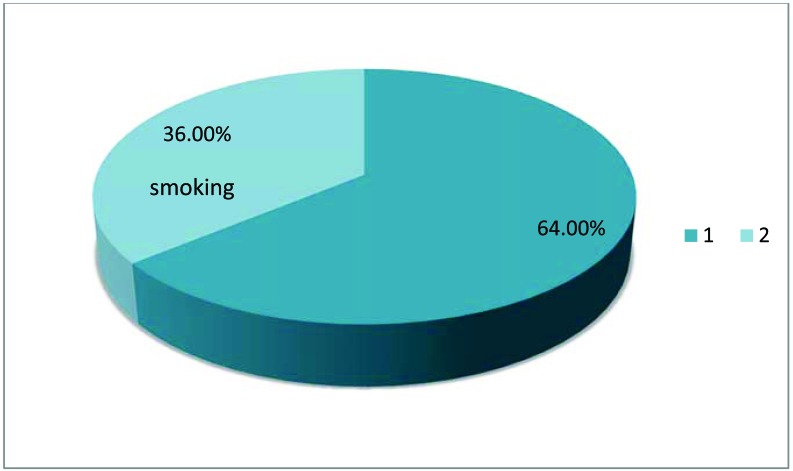
The prevalence of smoking in the spondyolarthritic patients of our series.

**Table 1:** Association between clinical characteristics Of Sa and tabacco.

Characteristic of AS			Smoking + n=77	Smoking- n=137	p
Average age (years)			38.84	40.29	0.618
Sex		M F	75(35%) 2(1%)	55(25 .7%) 82(38.3%)	≤0.0001
Term evolution		Average ≤2 >2	8.77±7.06 60(66.7%) 17(27.4%)	7.53±5.86 41(33.3%) 96 (72.6%)	0.176
Disease activity	BASDAI	≤4 >4	28(13.1%) 49(22.9%)	43(20.1%) 94(43.9%)	0 .004
	ASDAS CRP	Low medium high	10(1.6%) 16(16.1%) 51(82.3%)	12(6.5%) 14(11.4%) 111(82.1%)	0.036
Inflammatory systemic syndrome		YES NO	33(15.4%) 44(20.6%)	35(16.4%) 102(47.7%)	0.023
Severity of disease		YES NO	59(27.6%) 18(8.4%)	69(32.2%) 68(31.8%)	≤0.001
Functional impairment		BASFI≥4 BASFI<4	54(25.2%) 25(10.7%)	59(27.6%) 78 (36.4%)	0.038
Cervical stiffness		YES NO	46(21.5%) 31(14.5%)	71(33.2%) 66(30.8%)	0.031
Back stiffness		YES NO	43(20.1%) 34 (15.9%)	52(24.3%) 84(39.7%)	0.009
Lumbar stiffness		YES NO	63(29.4%) 14(6.5%)	98(45.8%) 39(18.2%)	0.064
ESR (1st Hour)			37,61	36,63	0.824
CRP			36,63	25,20	0.084.

AS:Axial Spondyloarthritis, BASDAI: Bath Ankylosing Spondylitis Disease Activity Index. ASDAS: Ankylosing Spondylitis Disease Activity Score functional effect .BASFI: Bath Ankylosing Spondylitis Functional Index.

**Table 2:** Association between factors severity of as and Tobacco.

Severity Factor of AS		Tobacco + n=77	Tobacco + n=137	p
young age of onset ≤ 16 ans	YES NON	32(40.3%) 45(59.7%)	48(33.3%) 89(66.7%)	0.219
Respiratory disease	YES NON	15(12.9%) 62(87.1%)	18(8.9%) 119(91.1%)	0.276
Uveitis	YES NON	16(12/9%) 61(87.1%)	22(12.2%) 115(87.8%)	0.531
Uveitis	YES NON	29(35.5%) 48(64.5%)	34(22%) 103(76%)	0.038
Inflammatory systemic syndrome	YES NON	33(43 .5%) 44(56.5%)	35(24.4%) 102(75.6%)	0.047
Resistance to NSAIDs	YES NON	31(38.7%) 46(61.3%)	57(40.7%) 80(59 .3%)	0.463

NSAIDs: Non-steroidal anti-inflammatory drug

AS: Axial spondyloarthritis

**Table 3:** Logistic regression.

Variable	OR	[IC] 95%	P
Sexe masculine	80.90	17,95 – 364,64	p=≤0.001
Disease activity (BASDAI)	2.545	0.973 ─6.660	P=0.059
Functional impairment BASFI	2.357	0.935 ─ 5.941	p=0.06
Severity of the disease	6.773	1,248 ─ 36,754	p= 0.027

BASDAI: Bath Ankylosing Spondylitis Disease Activity Index. ASDAS: Ankylosing Spondylitis Disease Activity Score functional effect .BASFI: Bath Ankylosing Spondylitis Functional Index.
